# Photonic Quantum Networks formed from NV^−^ centers

**DOI:** 10.1038/srep26284

**Published:** 2016-05-24

**Authors:** Kae Nemoto, Michael Trupke, Simon J. Devitt, Burkhard Scharfenberger, Kathrin Buczak, Jörg Schmiedmayer, William J. Munro

**Affiliations:** 1 National Institute of Informatics, 2-1-2 Hitotsubashi, Chiyoda-ku, Tokyo 101-8430, Japan; 2 Vienna Center for Quantum Science and Technology, Atominstitut, TU Wien, 1020 Vienna, Austria; 3 NTT Basic Research Laboratories, NTT Corporation, 3-1 Morinosato-Wakamiya, Atsugi, Kanagawa 243-0198, Japan

## Abstract

In this article we present a simple repeater scheme based on the negatively-charged nitrogen vacancy centre in diamond. Each repeater node is built from modules comprising an optical cavity containing a single NV^−^, with one nuclear spin from ^15^N as quantum memory. The module uses only deterministic processes and interactions to achieve high fidelity operations (>99%), and modules are connected by optical fiber. In the repeater node architecture, the processes between modules by photons can be in principle deterministic, however current limitations on optical components lead the processes to be probabilistic but heralded. Our resource-modest repeater architecture contains two modules at each node, and the repeater nodes are then connected by entangled photon pairs. We discuss the performance of such a quantum repeater network with modest resources and then incorporate more resource-intense strategies step by step. Our architecture should allow large-scale quantum information networks with existing or near future technology.

The development of devices that process information according to the principles of quantum mechanics is leading to a new technological revolution^1^,^2^,^3^,^4^. It is already clear that large scale quantum computers will be able to perform tasks impossible in the classical world, however it is a daunting tasks to realise due to the huge number of physical qubits (billions at least) required^5^,^6^,^7^,^8^,^9^,^10^,^11^,^12^. The field of quantum communication is rapidly growing as it is seen as a task simpler than full large scale quantum computation^13^,^14^,^15^,^16^,^17^,^18^. Even though it is likely that resources of similar quality will be required, much fewer of them will be needed^19^,^20^.

A key ingredient in any quantum communication network is quantum repeaters–devices that create entangled qubits between distance parties. The field has been working in two main directions: the first being the experimental realisation of small scale devices using high error rate components that unfortunately leads to poor communication performance^16^,^17^,^18^,^19^,^20^,^21^. The second has been theoretical work on large scale fully error corrected quantum systems, whose performance can be exceptionally fast but imposes quite demanding (and still to be realised) requirements on the physical hardware^19^,^20^,^21^,^22^,^23^,^24^,^25^,^26^. However, small scale quantum computers required in such quantum communication systems are far from what can be realised with current technology. One must bridge this gap to provide a viable route forward.

There are many potential mechanisms to distribute entanglement remotely including ones based on emitters, transmitters, receivers, and scatterers^14^,^21^,^27^,^28^,^29^. In this manuscript we illustrate how one can utilise the components and techniques being developed for large scale quantum computers in simple quantum repeaters–without initially having to resort to fully error-corrected devices. Our approach is based on the state dependent reflection of an optical photon^30^,^31^,^32^,^33^,^34^,^35^ interacting with a negatively-charged nitrogen vacancy center (NV^−^) in diamond embedded in a cavity^36^. The scheme we present in this paper does not restrict its implementation to NV^−^ centers in diamond. However a number of good quantum properties and the required controllability with NV^−^ centers have been demonstrated^37^,^38^,^39^,^40^,^41^,^42^,^43^,^44^,^45^,^46^,^47^,^48^,^49^. Further, entanglement between remote NV^−^ centers^50^,^51^ has recently been shown, and they are therefore promising candidates to implement such schemes.

The electron spin of an NV^−^ center is used to mediate entanglement between optical photons (without direct excitation) and the nuclear spin-1/2 of ^15^N which is used as the long-lived memory, while optical signals propagate between nodes. The same components can be used both for the entanglement distribution as well as the local two qubits gates. We however need to remember that NV^−^ centers feature an optical transition at 637 nm but telecom wavelengths need to be used over optical fibers. This necessitates the use of frequency converters–to or from visible to telecom wavelengths^52^.

In the following we first describe the basic components of the repeater, what is needed to build a linear network and discuss its performance. We then show how to boost performance by adding identical modules for multiplexing and error correction.

## The Module

**Figure 1:**
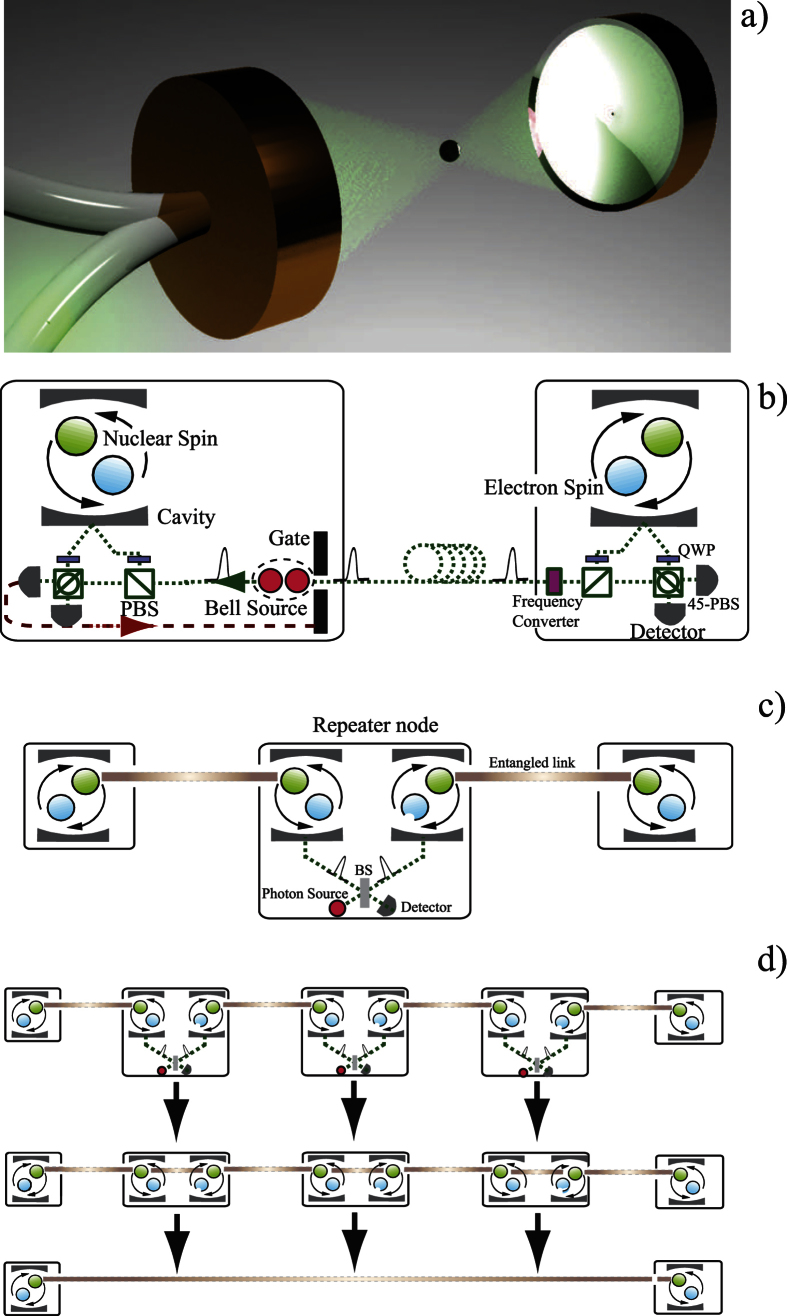
(**a**) Artistic impression of a quantum module consisting of an optical cavity and a single NV centre (NV^−^) in diamond. (**b**) Schematic diagram of an entanglement generation between two remote nodes connected by fiber. The nodes are composed of a NV^−^ center embedded in a two sided optical cavity. Alice’s node (left hand side) contains a polarisation source of 
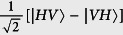
 entangled photons emitting one photon at the visible wavelength and the second in the telecom range, and a buffer/gating system to temporarily delay the telecom photon (The gate opens only upon a successful measurement event at Alice’s node). Bob’s node (right hand side) contains a frequency converter from the telecom to the visible wavelength. (**c**) Entanglement swapping within a repeater node based on the probabilistic entanglement of the individual NV^−^ electron spin using a single photon in a Mach Zender interferometer arrangement. Detection at the dark port implies a maximally entangled Bell state has been generated. Using the hyperfine interaction and electron spin measurements, the entanglement link can be transferred to the nuclear spins–effectively creating a chain of entangled nuclear spins^36^. (**d**) Entanglement swapping over many repeater nodes.

The most important component for the repeater is a (quantum) data processing module (depicted in Fig. 1a). We may require optical elements such as photon detectors, beam splitters, single photon and Bell state sources and coherent frequency converters. The module is an interface between photon and matter qubits which store and process the quantum data. We illustrate a design of such a module and its functions using a single NV^−^ center embedded in an optical cavity. The description given in this example can be applied to implement the same functions with other physical systems.

The module consists of an optical cavity and a single NV centre (NV^−^) in diamond^53^,^54^,^55^. The single NV^−^ centre provides an electron spin −1 and the nuclear spin −1/2 of ^15^N. The Hamiltonian of the single NV^−^ centre^36^,^47^ is


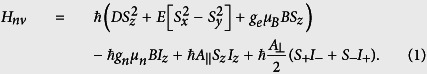


Here, the first term represents a zero field splitting (*D*/2*π* = 2.87 GHz), a strain induced splitting (*E*/2*π *< 10 MHz), and a magnetic field induced splitting (*g*_*e*_*μ*_*B*_*B*) for the NV^−^ centre’s electron spin^56^. *S*_*x*,*y*,*z*_ represents the generalised Pauli *X, Y, Z* operators for a spin-1 system with *S*_+_ (*S*_−_) being the raising (lowering) operator. The parameter *μ*_*B*_ is the Bohr magneton, and *g*_*e*_ = 2.0 is the electronic g-factor. With an externally applied magnetic field of *B* ~ 20 mT, the |0〉 and |+1〉 levels at the ground manifold are separated by approximately 3.43 GHz. The |*m*_*s*_ = −1〉 energy level is far detuned approximately 1.1 GHz below the |*m*_*s*_ = +1〉 level and ~2.3 GHz above the |*m*_*s*_ = 0〉 level. The electron states |0〉 and |+1〉 in the ground state manifold span the Hilbert space of the electron spin qubit in the module. The second term represents a magnetic field induced splitting of the nuclear spin of ^15^N. *I*_*z*_ is the Pauli *Z* spin-1/2 operator, *μ*_*n*_ is the nuclear magneton, and *g*_*n*_ = −0.566 represents the nuclear g-factor. The computational basis states of the nuclear spin are |↓〉 (|↑〉). The rest of the terms represents a hyperfine interaction between the electron and nuclear spins. The hyperfine interaction has both an Ising coupling with a coupling strength 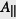 and an exchange coupling with a coupling constant *A*_⊥_. For the ^15^N nucleus these are 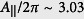 MHz and 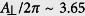 MHz respectively^56^.

Next we turn to the cavity and the NV^−^ electron spin. We tune the cavity to be resonant to the energy gap between |0〉 states of the ground and the first excited state |*M*_3_〉^36^. The electron spin states |0〉 and |+1〉 are used to conditionally reflect an incoming light field. Assuming a high-cooperativity regime for the cavity, i.e. *C* ≫ 1, a photon impinging on a cavity containing an NV^−^ centre in the |0〉 ground state is reflected (*P*_*r*_ ~ 1), while the signal for the empty cavity results in *P*_*r*_ ~ 0 36,57. We note that the required cooperativity for fault-tolerance in quantum computation is on the order of *C* ~ 20. Furthermore, recent advances in suitable microcavities indicate that the creation of large numbers of NV-cavity systems with this level of performance is realistically achievable^58^,^59^. In this regime, the procedure results in a quantum non-demolition measurement of the electron spin^60^. There are several advantages to this approach: In the vast majority of attempts, the photon does not enter the cavity and can therefore not excite the NV centre. The NV centre is also not used as an emitter, reducing the importance of photon extraction efficiency from the cavity. Furthermore, the use of single photons fully eliminates the possibility of spectral diffusion caused by ionization, as charge state conversion in single NV centres occurs via a two-photon process. In this regime, the electron state excitation decreases with cooperativity as 1/*C*, however there is a small possibility that an off-resonant excitation of the NV^−^ centre in the |+1〉 ground state may occur. In most cases, an excitation is harmless, however due to a number of decay channels with non-zero probabilities, it could cause a spin flip or leakage out of the qubit subspace into the |−1〉 ground state. Such an error could be transferred to the nuclear spin through the hyperfine coupling. Hence when dealing with photons, we have to assume that it is inevitable for any gates mediated with photons to be probabilistic, and hence we need to design the gate to be heralded for success. The heralding signal is given by the photon measurement, which guarantees that there was no excitation occurred in the case of success. When the gate fails, we need to treat the electron and nuclear spins carefully. If we can initialise both electron and nuclear spins after a failed event, it is straightforward to correct such errors. We can treat the entanglement distribution between adjoint nodes in this way, however in general we have to repeat the gate sequence until success while the nuclear spin carries nontrivial quantum information, and hence such errors can be accumulated. To deal with such errors, it has been shown that the use of an appropriately polarised optical field would be sufficient to suppress the unwanted excitations to meet the overall error rate for fault-tolerant quantum computation. Further features of the projective electron entanglement and the electron readout have also been discussed in previous work^36^.

The electron spin coherence times of single NV centers in isotopically purified CVD diamond have been measured near 1 s for *T*_1_ in the 4–80 K regime^61^, 90 μs for 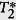, 2–15 ms for *T*_2_^62^,^63^,^64^ (*T*_2_ ~ 0.5 s for ensembles using dynamical decoupling techniques^65^), while the communication time for 100 km through a fiber is approximately 500 μs. Hence the electron spin coherent time may not be long enough to maintain the quantum information at fault tolerant levels. The coherence time for the nuclear spin of the ^15^NV^−^ centre is likely to be near 1 s or longer, which would be two orders of magnitude longer than the single electron spin coherence times^37^. Hence the nuclear spin may be used as a quantum buffer or memory instead. The hyperfine coupling can be used to realise a CZ gate between the electron spin and the nuclear spin^36^,^66^. The hyperfine interaction is always on, which could be a decoherence source for the nuclear spin, however we can effectively turn off the hyperfine coupling by setting the electron state to be |0〉.

Now, we turn to single qubit rotations and measurements, which are essential for initialisation, single-qubit operation, and readout. We start with spin rotations. The spin rotations can be implemented via an electromagnetic driving field, the interaction can be given as


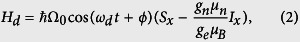


where Ω_0_ is the amplitude of the applied field. The frequency *ω*_*d*_ is chosen to determine whether we drive the electron or nuclear spin with the specific phase *ϕ*. The initialisation and readout can be done through measurements. The projective measurement of the electron spin in the computational basis can be implemented as QND measurement via photon. The conditional reflection introduced previously can be used to determine whether the electron spin state is |0〉 or |1〉 by detecting the reflected photons. Combining it with single rotations, we can implement *X*− and *Y*− measurements. Such QND measurements can also be performed using weak coherent states, however to achieve a higher overall efficiency, we use repeated QND measurement with single photons. This way we avoid deionisation of the NV^−^ and also minimise unwanted excitations to the first excitation manifold^67^,^68^. There is a small possibility that the electron spin state leaks to |−1〉. Through the repeated QND measurement, we can detect such leakage and reset the electron spin by the spin rotation. The projective measurement on the nuclear spin can implemented via the hyperfine interaction and the QND measurement on the electron spin^36^. The natural hyperfine interaction enables fast *Z*− basis measurements, while *X*− and *Y*− basis measurements can be done with a driven hyperfine interaction^66^. Combining the measurement schemes and the single qubit rotations, we can initialise both the state of the electron and nuclear spins.

As we described above, the NV^−^ centre in an optical cavity is a good candidate to construct the module, however this is not only the implementation possible. As long as all the module functions are satisfied with required fidelities, a design radically different from this may be considered. The specific physical parameters are necessary for the construction and evaluation of the module and its systems^36^.

## Remote Entanglement Distribution

Our scheme for the remote entanglement distribution is depicted in Fig. (1b). This scheme is applied to establish entanglement between two adjoint nodes. As we mentioned above, the cavity of the module is tuned to conditionally reflect an incoming photon only when the electron spin state is |0〉, and we use this conditional reflection to establish entanglement between two electron spins in different modules^36^. As shown in Fig. (1b), a high-rate Bell source as well as a polarisation selective detector is inserted at the sender node. The creation of the link begins by preparing the NV^−^ centres electron spin in the state 
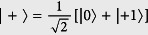
 and by the Bell source emitting an entangled pair, 
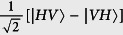
, with one photon at the telecom wavelength and the second at the wavelength of the NV^−^ center in the visible regime (see ref. 69 as an illustrative 800/1550 nm example). The telecom photon, to be sent to the receiver node, is temporally buffered while the visible photon is split on a polarising beamsplitter into two modes, one containing the horizontal polarisation component and the second the vertical component. This vertical component is then rotated by a quarter wave plate to *σ*_+_ polarisation where it interacts with the cavity containing the NV^−^ center. A *π*-phase shift is applied to the incident photon if the NV^−^ center was in |0〉 state, while it is transmitted through the cavity and lost if the NV^−^ center was in the |+1〉 state^36^. The reflected photon is then rotated back to vertical polarisation and combined with the horizontal components on a 45° PBS. The photon is then measured in the diagonal (D) basis giving a result *D, A* or 0, where 0 indicates no detection event.

**Figure 2:**
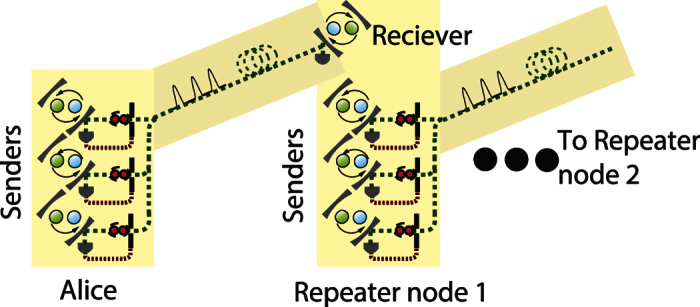
Schematic diagram of a spatially asymmetric multiplexed quantum repeater scheme^19^.

In the event of a photon detection, the electron spin in the sender module is entangled with the telecom photon stored in the buffer. We underline that the long fiber link between distant nodes can act as the buffer. Now, two independent operations can occur at the same time. In the first we release the stored photon into the channel (converted to time bin encoding) and second we transfer the state of the electron spin to the nuclear spin whose coherence time is significantly longer than that of the electron spins, so avoiding the deterioration of the fidelity from the electron spin dephasing. This entanglement transfer operation can be done via the hyperfine coupling, the electron spin measurement, and initialisation. The photon released into the channel is transmitted over the link to the adjacent repeater node (along with the measurement result from the first photon). Upon arrival of the photon at the remote receiving node, two sequential operations occur, first the time bin encoding is transformed back to polarisation and then its frequency is converted to the optical wavelengths and a polarising beamsplitter separates the vertical and horizontal polarisations. The vertical polarisation mode is rotated to *σ*_+_ polarisation and then interacts with the second NV^−^ center also prepared in the |+〉 state. Again a *π*-phase shift occurs on this photon if the electron spin was in the |0〉 state (scattered for the |+1〉 state). As previously the photon is recombined on a PBS and then measured in the diagonal basis. When the photon is successfully measured, the entanglement is stored in the nuclear spin, again via the hyperfine coupling and the electron spin state measurement. The resulting state, dependent on a successful measurement result, is


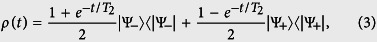


for the *D, A* and *A, D* events with 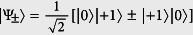 and a fidelity 
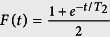
. Here *T*_2_ is the coherence time of the nuclear spins while *T*_*R*_ (*T*_*R*_ ≪ *T*_2_) is the roundtrip time for a signal to propagate between adjacent nodes [In this situation we assume the central node in Fig. (2) perform entanglement swapping as soon as the links to the left and right are established. No waiting time is required for the memories in this case assuming one is careful in arranging how the links are connected. However the left and right outer nodes have to wait on the roundtrip time *T*_*R*_ before they can use their resources. Hence one roundtrip time of dephasing on the nuclear spins arises]. Each of these events (D, A, A, D) occurs with a probability 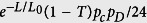, thus giving an overall success probability


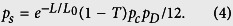


Here *L* is the length of the channel between the nodes, *L*_0_ the attenuation length of the fiber, *p*_*D*_ the single photon detection probability, *p*_*c*_ the coupling efficiencies associated with the cavity interaction (including the frequency conversion efficiency) and *T* the transmission coefficient of the cavity path (*T *~ −1) [Generally in entangled photon based schemes, the probability of success scales as 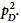 However as pointed out by Jones *et al*.^70^ this can be reduced to scale as *p*_*D*_ by using high rate Bell sources and conditioning. This requires that the rate at which measurements can be performed in the local node is sufficiently fast such that a successful result occurs in a time much shorter than the transmission time between nodes. Overall the success probability *p*_*s*_ is the same scaling one would expect from the single photon scheme when the source efficiency is 100%. Further as the overall scheme is based coincidence detection, it is robust against dark counts]. If the detection event was 0, the procedure has failed and the protocol needs to start again. At this stage of the entanglement generation, there is no communication information involved in the procedure, and hence the electron and the nuclear spins can be measured and reinitialised for the next round of entanglement generation without any loss of communication information.

## Performance and Rates

Each attempt of the entanglement generation described above is probabilistic in nature, but can effectively be made deterministic (or near deterministic) in nature using spatial or temporal resources. With limited physical resources, a spatial approach is not available and so a temporal approach (a repeat until success strategy) must be used sacrificing the operational time. To achieve a failure probability 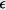 for the basic entanglement link, 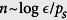 attempts are required. After the *n*^*th*^ attempt, the missing link probability 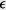 reduces the rate of the basic entanglement link to


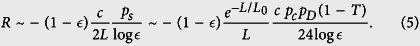


We could have the rate *R* to be 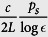 incorporating the failure factor 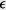 to the fidelity of the Bell pair. However, with the heralded signal, we know when the link failed, and hence we can keep the fidelity untouched sacrificing the generation rate.

## Simple Linear Chains

The next step is to move from two adjoint nodes to a linear chain of quantum repeater nodes. Among a number of approaches and strategies that can be used to implement a linear chain quantum repeater, the minimal resource approach by Childress *et al*.^14^ uses one NV^−^ center per node hosting two qubits (an electron spin and a nuclear spin). However there are disadvantages to this in that the electron spins coherence time significantly limits the quality of the longer range entangled links that can be constructed. Also, long electron-nuclear interactions can lead to contamination of the information on the nuclear spin. Instead by using two NV^−^ centers per node (Fig. 1c), one can use the nuclear spins’ long coherence properties to establish all the long range link, yet use the electron spins for interface to distribute entanglement both within and between the nodes. The local two qubit gates between NV^−^ centers in different cavities within the same node are mediated via an optical link which facilitates the two electron spins to be entangled. This electron spin entanglement can then be transferred to the nuclear spins creating an entanglement chain, i.e. a linear cluster state, of NV^−^ center electron and nuclear spins^36^. By measuring the nuclear spins in the intermediate repeater nodes in an *X* basis, they are disentangled and a longer range nuclear spin entangled link created (Fig. 1d).

**Figure 3:**
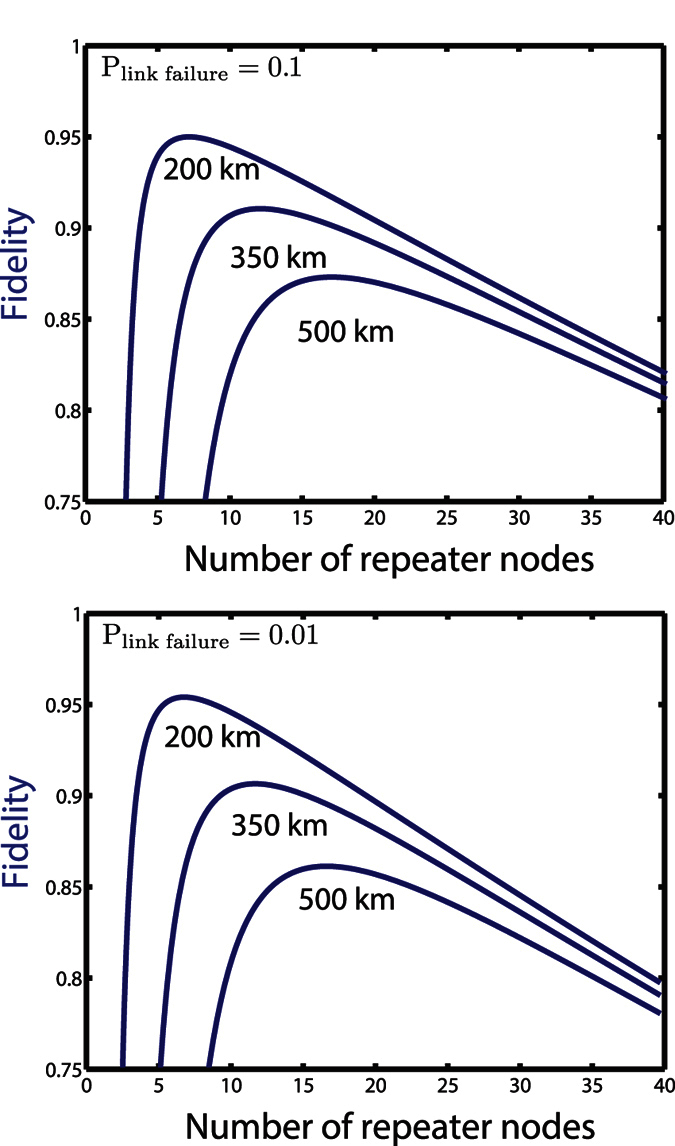
Fidelity of the resulting Bell state between the end node of the repeater chain versus *N* for network distances of 200, 350 & 500 km.

Given the high initial fidelity of the nuclear spin links and the near deterministic local gates, we can now estimate the performance of such a repeater scheme. One can also easily establish an estimate for the rate to generate Bell pairs between the end nodes in the linear chain as


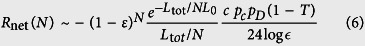


where *N* + 1 is the number of repeater nodes in the chain. The expression of *R*_n*et*_ indicates that increasing the number of repeaters nodes (increasing N) for the fixed distance *L*_*tot*_ would give a better performance, however as our initial entangled links have a finite fidelity *F*, performing *N*−1 entanglement swapping operations would lead to a decrease in the overall fidelity 
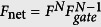
, where *F*_*gate*_ is the gate fidelity for performing swapping operations. Increasing the number of nodes degrades the fidelity. To illustrate this we plot the resulting fidelity in Fig. (3) versus *N* for a 200 km, 350 km & 500 km network. The initial, high fidelity links between adjacent repeater nodes plus fast, near deterministic and efficient local gates indicate that purification is not required as long as the number of nodes is small. For the smaller number of nodes, the probability of success for a single link generation between adjacent nodes is quite low and so many attempts are needed (>100). Fewer nodes require less entanglement swapping gates, but the time required to generate an end-to-end link eventually approaches the lifetime of the nuclear spin, which then limits our Bell pair fidelity. This limiting factor causes the sharp drop in fidelity visible in Fig. 3 when the number of nodes is decreased below its optimal value. For large *N*, the performance is instead limited by the gate infidelity *F_gate_*.

An issue arises where one wants to make comparisons with different numbers of repeater nodes. Changing the number of nodes or the number of qubits within a node will dramatically change its rate of communication. Hence some form of resource normalisation could be appropriate. There are many ways this could be achieved, but a natural one would be to divide the rate by the total number of qubits in the whole network^26^. In such a case this normalised rate 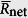 can be estimated as


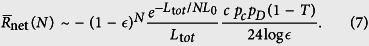


## Secret Key Rate

**Figure 4:**
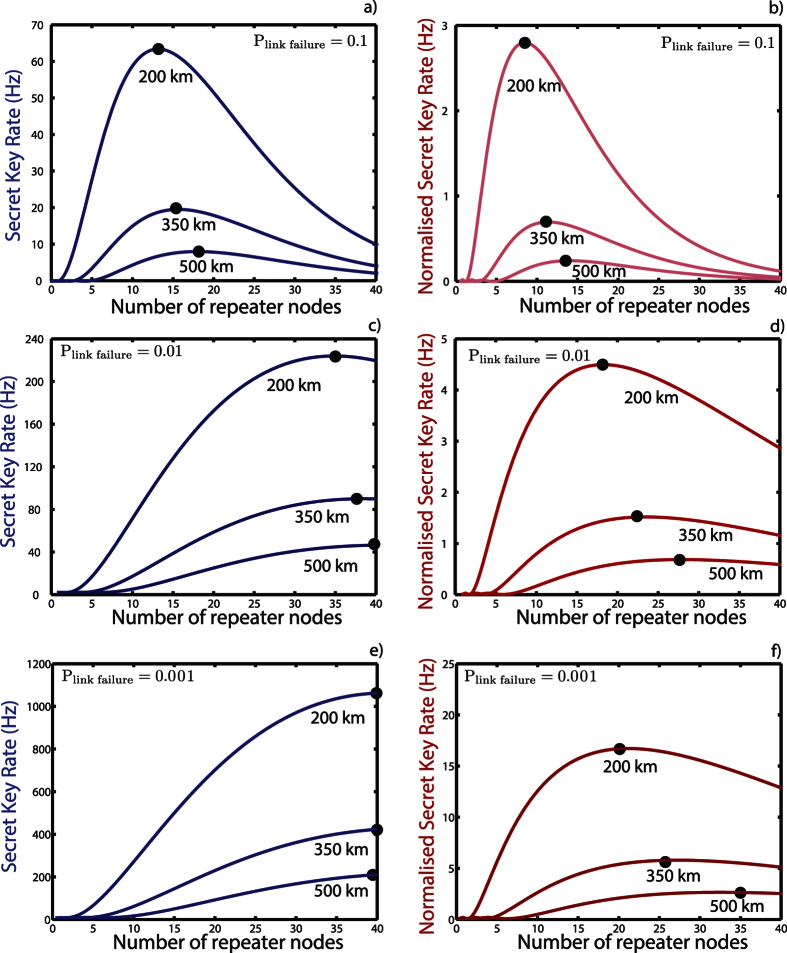
Long distance secret key rates (**a**,**c**,**e**) and normalised secret key rates (**b**,**d**,**f**) for an *N* + 1 node linear repeater network with total distances of 200, 350 & 500 km for various link failure probabilities *p*_link failure_ = 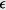 = 0.1, 0.01, 0.001. The network has *N* − 1 intermediate repeater nodes with each node containing two NV^−^ centers. The secure key rate is calculated by multiplying the end to end link rate by 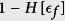 where *H*[*x*] is the binary entropy function and 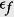 the error rate of the end to end Bell pair. The normalised secure key rate is calculated by multiplying the end to end link rate by 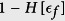 divided by the total number of NV^−^ centers used in the network. In (**c**) with *p*_link failure_ = 0.01, the optimal separation between the repeaters nodes are 5.71 km, 9.21 km and 12.20 km respectively, while for (**d**) it is 11.11 km, 15.21 km and 17.86 km respectively.

Being able to determine the rate and fidelity of entangled state generated over the end points of the networks gives us very useful information about the performance of our scheme. As mentioned earlier one of the natural applications for a long range entangled link is quantum key distribution. In most QKD scheme the keys can only be established over a maximum distance of 200 km and at this extreme range, the rate is generally quite low^71^,^72^. Given our repeater scheme, we can now determine the secret key rate and normalised secret key rate by the number of devices one can generate over 200, 350 and 500 km. Our secret key rate *C*_*r*_ (normalised secret key rate 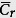) is given by the rate *R*_net_ (normalised rate 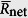) of generating entangled links multiplied by the factor of secret keys material per Bell state. This can be expressed as


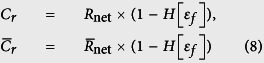


respectively where 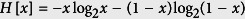 is the binary entropy function and 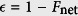 the error rate of the end to end Bell pair. This is due to the dominant phase error in the system, this simple entropy function is enough for our estimation. In Fig. (4) we plot the secret key and normalised secret key rate versus the number repeater links for three choices of the failure probability for a link being generated between adjacent nodes, 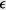
* *= 0.1,0.01, 0.001. Figure (4) shows the different performance for each 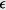. For 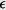 *=* 0.1 there is 10 percent chance an individual link will failure and in this case the overall chain failures. This means one does not want too many repeater nodes present (which can be seen from the peak maximum between 10 and 20). However for 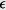 =* *0.001 the failure probability is on the order of 0.1 percent and so for *N* < 50 links do not fail very often. This means it should be rare for the entanglement chain to be broken but the cost will be that we wait a long time for the individual nodes between adjacent repeater nodes to be generated and so the overall rate could be low.

An 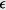 value between these should have better performance which can be seen in Fig. (4c,d). What is quite interesting is that the optimal number of repeater nodes depends heavily on whether one is considering the raw or normalised rates. As we add extra nodes to the repeater chain, the raw rate can obviously increase until the loss in fidelity balances it out. However for the normalised rate, we also need to divide by the 2*N* NV^−^ centers used in the linear network and so we would expect the optimal point to be reached for fewer repeater nodes. Thus we expect the number of nodes to be less in this second case.

The low key rate for small numbers of repeater nodes is again due to the probability of success for each try of a single link between adjacent nodes being quite low, and hence many attempts are needed. This means the time to generate our end to end entangled links starts to approach the life time of the nuclear spin and so the fidelity of the resulting state is low. For large *N* the fidelity is also limited by the 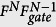 fidelity. These two effects compete with one another, so there is an optimal point at moderate *N*. A repeater distance between 10 km and 20 km works well for the assumed parameters.

## Multiplexing

Our performance for creating longer range links is primarily limited by the time to create the entangled links between adjacent nodes, and not by the local gates to perform the swapping operations. When the node separation exceeds the attenuation length of the fiber, we need to wait a significant number of round-trip times for quantum/classical signals to be sent between adjacent nodes. This is a form of temporal multiplexing but it also has a secondary detrimental effect on our NV^−^ centers due to their finite coherence times. This memory issue can be overcome by using a spatial multiplexing strategy costing more physical resources. However a more efficient spatial strategy can increase the performance further^19^, as we depict in Fig. (2), as it uses approximately half the resources of the previous schemes.

With *n* senders and 1 receiver, the probability a link is established is *p*_*S*_ = 1−(1 − *p*_*s*_)^*n*^. Of course more than one link between adjacent repeater nodes can be established at the same time. In fact, if one requires *q* copies then the success probability for *n* senders is 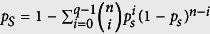. These *q* copies can be used in a number of ways including:

- Increasing the rate for generating long range Bell states.
- Performing some form of error correction to increase the range and fidelity of the long range Bell states.

**Figure 5:**
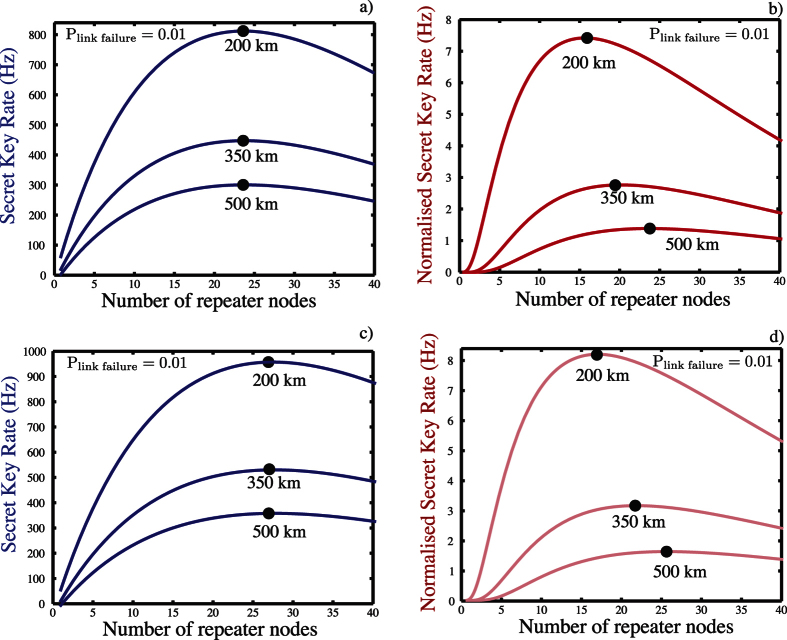
Long distance secret key rate (**a**) and normalised secret key rate (**b**) for an *N* + 1 node linear repeater network with total distances of 200, 350 & 500 km for a link failure probabilities *p*_link failure_ = 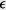 = 0.01. The network has *N* − 1 intermediate repeater nodes with each node containing *m* + 1 NV^−^ centers. In (**a**) the optimal separation between the repeaters nodes are 8.33 km, 14.58 km and 20.83 km respectively for the 200, 350 & 500 km overall distances, while for (**b**) it is 12.5 km, 16.67 km and 20.83 km respectively. Similarly, (**c**,**d**) are the long distance secret key rate and normalised secret key rate, respectively, for an *N* + 1 node linear repeater network when more than 1 qubit of information is encoded on the photon. The network has *N* − 1 intermediate repeater nodes with each node containing multiple NV^−^ centers. In (**c**) the optimal separations between the repeaters nodes are 7.41 km, 12.5 km and 17.86 km, respectively, for the 200, 350 & 500 km overall distances, while for (**d**) it is 11.76 km, 15.91 km and 19.23 km respectively.

Let us consider the first item. In Fig. (5a,b) we show the long distance secret key rate and normalised secret key rate for several network lengths, using the multiplexed strategy depicted in Fig. (2). Within a repeater node we have *n* + 1 qubits, *n* qubits used to establish entanglement to its right hand neighbour and one to act as a receiver to accept connections coming from the left hand adjacent repeater node. Of course as we are using more qubits per node, our raw (un normalised) secret key rate increases. The normalised rate however also increases and this is primarily due to removing the detrimental memory effects.

The second case is quite interesting as it opens another possibility for how we send information between the nodes. We could now encode multiple bits of information (say *q* bits) onto the transmitted single photon using time-bins, frequencies etc. In such a case we create a Bell state composed of the NV^−^ center (one qubit) and a hyper-encoded photonic state (encoded qubit). This hyper-encoded photonic state is transmitted over the network to the receiver side where its state is transferred back to *d* qubits, and decoded to correct errors that had occurred during the transmission or on the receiver side (It however will not correct memory based errors on the original bare NV^−^ center qubit). The hyper-encoding allows us to improve the key generation rate as is shown in Fig. (5c,d). However such a strategy does not allow us to significantly increase the total distance entanglement can be generated over, due to the imperfect Bell pairs created between adjacent nodes and errors associated with the local gates (~0.3%). It is difficult to have a normalised key rate greater than 1 bit/s for distance greater than 1000 km.

## Going longer: Error correction

To establish longer links one needs to perform either long range purification or error correction. Usual pair-wise purification is not ideal as it requires extensive classical communication which significantly limits its performance and the extensive classical communication dramatically increases the requirements on the quantum memories^73^. Error correction could exhibit similar limitations, however error correction provides different ways to protect the coherence of the state^74^, and hence its use does raise a number of important issues. First and foremost is the effect on performance by doing the error correction itself. To perform error correction we need many entangled links between adjacent nodes and so one would think that the rate of communication would decrease. For a distance *d* error correction code, *n*_*d*_ entangled pairs are needed. It is straightforward to show the rate for generating an encoded entangled link over a distance of *L*_total_ = *NL* divided by the total number of qubits used in the network is


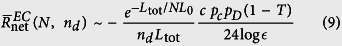


**Figure 6:**
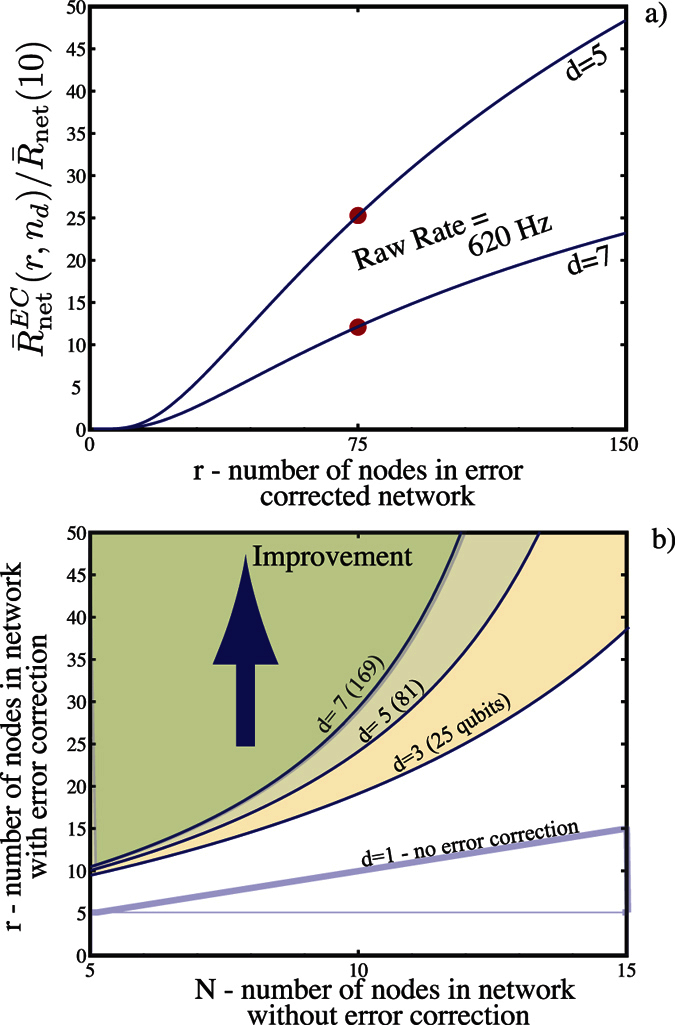
(**a**) Normalised rate 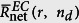 (for a 2000 km fully error corrected network (with codes distances of d = 5 and d = 7) compared with the rate 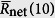 of a 10 link repeater network without error correction. The raw communication rate is 620 Hz for both the d = 5 and 7 codes. Both achieve a final fidelity for their entangled pair grater than 99% (99.9% and 99.99% respectively). The fidelity of the straight (no error correction) 10 link network is approximately 92%. (**b**) Comparison of the rate of generating an entangled link over a 2000 km network using various topological error correction codes of distance *d* = 3,5,7 (with qubit numbers 25,81,169 respectively). Here *N* + 1 is the number of nodes in the case of no error correction while *r* is the number of nodes in the error corrected case. Improvement in the normalised rates are shown as shaded areas above the corresponding *d* boundary.

We immediately notice that the normalised rate is lower by a factor of *n*_*d*_ compared to the uncorrected case. However this does not mean that the error correction case can not give us an improved normalised rate. To show this quantitatively, we assume a 10 link linear quantum repeater over 2000 km, which gives the normalised rate 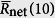, and see if there is any strategy with error correction of the normalized rate 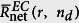 that can exceed 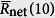. In Fig. (6a) we compare the performance using normalised rates of two error corrected codes with distances of d = 5 and 7, which are based on topological codes, requiring *n*_*q*_ = 81 and 169 respectively qubits per node in this scenario. The plots clearly show that once the number of nodes *r* > 20, a rate improvement can be obtained. More specifically for 75 nodes, the improvement is 45 times for the d = 5 code and 14 times for the d = 7 code. The d = 7 code however does give a much higher fidelity pair than the d = 5 case (by at least one order of magnitude). This does lead to a natural question of what the requirements are for an improvement in rate.

## Improvement Criteria

Now we formulate the criteria for improvement by error correction. For 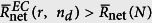 we require


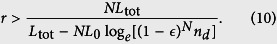


An improvement can therefore only be obtained when 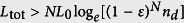 and *r* > *N*. Further if the separation between nodes *L* = *L*_tot_/*N* is less than the attenuation length of the fiber *L*_0_, error correction can not increase the performance. To illustrate this we show in Fig. (6b) the boundaries for different *n*_*d*_ with *L*_t*ot*_ = 2000 km where 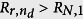. Above these boundaries an overall improvement to the normalised rate occurs.

## Discussion

We have presented a simple repeater scheme based on NV^−^ centers in diamond which can be used for a few node network, yet scaled to a large scale networks as more resources become available. For these shorter distances between 200–500 km, our simple two NV^−^ centers per node scheme can give significant gain in secret key rate compared to a direct transmission approach. Although such a setting is not optimal, a repeater network composed of a total of ten NV^−^ centers can exhibit a significant improvement over the 200 km range. For longer distances, the performance (Fig. 4) shows the trade off between the longer waiting time for entanglement distribution in two adjoint nodes and the cost associated with the larger numbers of swap operations when we have more repeaters stations. Such a simple scheme does not have a mechanism to improve the waiting time nor to recover from the increasing gate errors. However it can be addressed by introducing multiplexing for the former and error correction for the latter. In principle spatial multiplexing allows us to reduce the waiting time to just the round trip communication time between adjacent nodes but at the cost of more resources per node. Error correction allows us to overcome both the errors coming from local swap gates as well failed links, however it imposes a significant resource overhead in terms of both the number of NV^−^ centers required and the number of links required between adjacent nodes. Error correction pushes up the fidelity of the final Bell-pair and so can help us achieve a better normalised secure key rate. For instance, error correction allows to achieve a 99.9% final fidelity Bell state with reasonable rate over 2000 km. This is sufficient for fault tolerant quantum computation and communication requiring a local gate error rate of ~10^−4^ for a 20 node linear repeater network. Finally our architecture can in principle allow for large-scale quantum information networks with existing technology but can be used immediately with a small number of nodes for practical quantum communication tasks.

## Additional Information

**How to cite this article**: Nemoto, K. *et al*. Photonic Quantum Networks formed from NV^−^ centers. *Sci. Rep.* **6**, 26284; doi: 10.1038/srep26284 (2016).
